# Health Care for Persistent Somatic Symptoms Across Europe: A Qualitative Evaluation of the EURONET-SOMA Expert Discussion

**DOI:** 10.3389/fpsyt.2018.00646

**Published:** 2018-12-07

**Authors:** Sebastian Kohlmann, Bernd Löwe, Meike C. Shedden-Mora

**Affiliations:** Department of Psychosomatic Medicine and Psychotherapy, University Medical Center Hamburg-Eppendorf, Hamburg, Germany

**Keywords:** persistent somatic symptoms, medically unexplained symptoms, somatoform disorders, somatic symptom disorders, functional syndromes, health care, Europe

## Abstract

**Background:** Persistent somatic symptoms (PSS), referred to as somatoform disorders and lately somatic symptom disorders, are frequent but often remain under-recognized and under-treated. Although European guidelines overlap, presumably, there is large diversity in their practical use and in the actual health care situation. The aim of this qualitative evaluation was to compare health care for PSS across 9 European countries, to illustrate commonalities and differences, and to discuss challenges for a pan-European research agenda.

**Methods:** A case vignette fulfilling ICD-10 criteria of undifferentiated somatization disorder was presented to 24 experts from 9 European countries, who completed a semi-structured assessment regarding the routine management including diagnostic procedures, treatment recommendations, and country-specific health care aspects. A qualitative evaluation was conducted using the video-transcripts of the presentations. Results were validated by additional expert interviews.

**Results:** Across all countries, primary care physicians serve as the gate keeper for further diagnostic and treatment procedures. Apart from this commonality, there is a large variability in health care routines. Experts concluded that individuals with PSS appear to be a non-identified patient group within many European health care systems. To overcome the gap between evidence-based guidelines and clinical reality needs, three key challenges were identified: (1) Defining a clinically useful, acceptable, and non-stigmatizing diagnostic term, (2) implementing guideline recommendations into routine care, (3) developing effective dissemination strategies.

**Conclusions:** The results advocate for more research on the actual European health care situation. A systematic European research agenda with unified goals and interdisciplinary collaboration that integrates all stakeholders could answer this challenge.

## Introduction

Across Europe, persistent somatic symptoms (PSS) constitute a frequent major health problem associated with substantial individual and societal burden. Referred to as somatoform disorders, medically unexplained symptoms, functional disorders, bodily distress and lately somatic symptom disorder, the 12-month prevalence rate is estimated 5% (i.e., 20 million individuals in Europe) (1). PSS are frequent across both primary and specialized health care settings (2, 3). While effective treatment exists (4), PSS frequently remain under-recognized and under-treated, with durations of untreated illness of up to 25 years (5). The clinical reality is often characterized by unstructured use of specialized care, resulting in high costs (6). Although national guideline recommendations in Europe overlap (7), there is presumably a heterogeneity in the actual health care situation across Europe. PSS result from the complex interplay of biomedical, psychological and social factors (4). This multifactorial etiology further complicates the deduction of a clear diagnostic and treatment rationale. Additionally, intercultural factors contribute to a diverse understanding of PSS (8).

The “European Research Network to improve Diagnosis, Treatment and Care for Patients with Persistent Somatic Symptoms” (EURONET-SOMA) aims to contribute to a common understanding of the terminology, etiology, conceptualization, and effective management approaches of PSS (9, 10). The EURONET-SOMA group consists of more than 36 international experts from 14 European countries who have met biannually since 2016. We aim to address the pressing needs for improvement in the management of patients with PSS within a joint research agenda. Yet, we face the challenge of conducting research and managing patients within very different health care systems on the one hand, but aiming to draw conclusions on an international level on the other. The aim of this study was to compare health care for PSS, to illustrate commonalities and differences, and to discuss challenges for a pan-European research agenda.

## Methods

**Figure 1:**
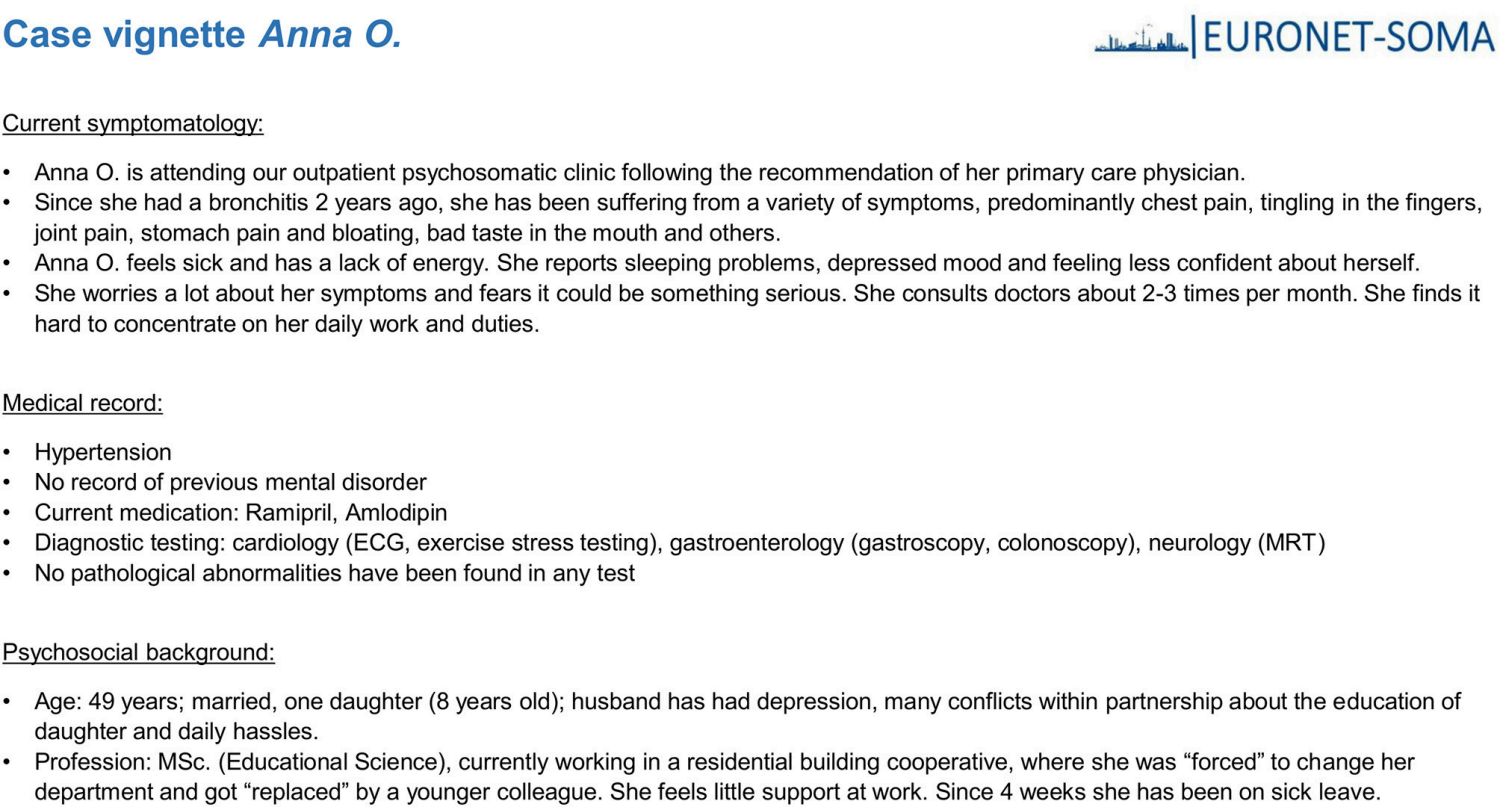
Patient vignette of Anna O. developed according to the ICD-10 criteria of undifferentiated somatization disorder.

During the fourth EURONET-SOMA meeting in Riga, Latvia in October 2017, 24 experienced researchers from 9 countries (Belgium, Denmark, Germany, Netherlands, Norway, Latvia, Poland, Sweden, United Kingdom) took part in the discussion. Among them were specialists from psychosomatic medicine, internal medicine, psychiatry, clinical psychology, and primary care. The majority of experts has years of expertise in the research and clinic of PSS. Experts received a patient vignette of Anna O. that was developed according to the ICD-10 criteria of undifferentiated somatization disorder (Figure 1). Country wise, experts completed a semi-structured assessment asking for the typical health care regarding typical terms used, diagnostic procedures (somatic, psychometric), availability of guidelines, first line recommendations, treatment (i.e., psychotherapy, pharmacological), and health care delivery. Additionally, experts were asked to provide a graphical depiction of the typical flow of this patient through their health care system. Experts presented their country-specific management of PSS to the panel. The session was video-taped. Afterwards, all experts discussed commonalities, differences, best-practice models, and challenges for a pan-European research agenda. Based on the semi-structured assessments and videos, two researchers (SK and MSM) independently coded the answers as present, partially present or not present. Divergent coding was discussed to reach agreement. Structured telephone interviews with an expert from each country (see acknowledgments) followed to compliment and validate the results. Additionally, all material was revised with respect to information not included in the deductive categories. Thereby, our approach followed qualitative analysis principles by identifying major themes and by integrating the information across countries.

## Results

Table 1 summarizes the main findings. Terms used for PSS largely vary among experts. Yet, there is a commonality across countries: Whereas mental health specialists use ICD/DSM terms (e.g., somatoform disorder), other health care providers [e.g., primary care physicians (PCPs)] code varying diagnoses ranging from syndrome-specific terms (e.g., chronic fatigue) to medically unexplained symptoms (MUS) or “somatically not enough explained bodily symptoms” (SOLK) in the Netherlands, to depression. Most experts noted that a term for PSS that is associated with a mental disorder would hardly be used in primary care. Across all countries, the major commonality included the role of the PCP as the first contact person and coordinator of diagnostic and treatment procedures. Yet, diagnostic and referral procedures appear to be inefficiently structured in clinical routine (i.e., loops from PCP to several somatic specialists and back) and by chance patients are referred to mental health care. If guidelines are available, their use in clinical practice is rare. Although most experts agreed on wisely choosing necessary tests, in clinical routine there is extensive somatic testing. In most countries, a conservative use of antidepressants is recommended but pain medication is commonly prescribed. Recommendations to initiate psychotherapy, as well as its content, length and payment policy largely vary. Specialized PSS treatment centers are rare, and rather focus on single functional syndromes. Although empirical knowledge is scarce, experts consented that complementary and alternative treatments for PSS appear to be often used by patients.

**Table 1:** Evaluation of EURONET-SOMA expert discussion regarding health care for persistent somatic symptoms in 9 European countries.

**Countries**	**BE**	**DK**	**GB**	**GER**	**LV**	**NL**	**NO**	**PL**	**SE**
**TYPICAL TERM USED**									
Functional disorder		X		X		(X)		
Somatoform disorder	X			X	X	(X)	(X)	X
No specific term/ varying terms								X	X
MUS/SOLK			X			X		
Depression					X		X	X
Stress related symptoms	X	X						
Undiagnosed biomedical problem							X	
**GUIDELINES PROPOSING STEPPED CARE**									
Available	(X)	X	(X)	X		X		
In progress								X	X
**SOMATIC TESTING**									
None						X		
Laboratory (blood, urine, endocrine)	X	X			X		X	X	X
Specific somatic testing	X	X			X			X	X
Conservative use of diagnostic tests		X	X	X	(X)		X	
**PSYCHOMETRIC TESTING**									
Generally recommended	(X)	X	X	X		X	X		X
Depression		X		X	X		X		X
Anxiety		X		X					X
Symptom burden						X			X
**FIRST LINE RECOMMENDATION**									
Primary care based management	X	X	X	X	X	X	X	X	X
Psychotherapy	X	X	X	X	(X)		X	X	X
Medication					(X)		X	X
**PSYCHOTHERAPY**									
Cognitive behavioral therapy	X	X	X	X	X	X	X		X
Psychodynamic therapy	X			X	(X)	(X)		X
Problem solving						(X)	X	
Acceptance-Commitment Therapy		X			X	(X)		
Paid by health insurance		(X)	X	X		X	X	X	X
Typical number of sessions	5–8	8–12	4	25–40	12–20	11–25	5–10	25	20–80
**MEDICATION**									
Antidepressants	X	X	X	X	X	X	X	X	X
Pain medication	X	X		X		X			X
Sleep medication					X			
**TREATMENT SETTING**									
Outpatient	X	X	X	X	X	X	X	X	X
Inpatient				X		(X)		

*BE, Belgium; DK, Denmark; GB, Great Britain; GER, Germany; LV, Lativa; NL, Netherlands; NO, Norway; PL, Poland; SE, Sweden; SOLK, Somatically not enough explained bodily symptoms; MUS, Medically unexplained symptoms. X, present; (X), partially present*.

## Discussion

Within the limitation of being non-exhaustive and non-systematic, this qualitative study among leading European experts points to the large variability in health care for patients with PSS. This results in three key challenges:

First, defining a clinically useful, acceptable, and non-stigmatizing diagnostic term. Persistent somatic symptoms emerged as a consensus “working term” within the EURONET-SOMA network and represents a starting point for further investigation (9). Second, implementing guideline recommendations into routine care within the diverse range of European health care systems. This also highlights that PSS needs to be covered as a diagnosis by health insurances and health policy. Third, developing effective dissemination strategies to actually reach patients with evidence-based approaches. Innovative examples addressing both clinicians and patients include mental health consultations in primary care, self-help, e-health interventions, and networks facilitating referral (11, 12). A central conclusion from our discussion was that individuals with PSS appear to be a non-identified patient group within many European health care systems: They rarely reach specialized care, but are widely treated by other health care providers and probably seek help outside the medical health care system (e.g., complementary and alternative medicine). Further research should focus on the early identification of PSS and, thus, avoid unnecessary somatic testing, which is common across different specialties. An integration of experts from other somatic medical disciplines (e.g., neurology, orthopedics, and immunology) into research networks such as the EURONET-SOMA could be one small step ahead. Certainly, a more systematic assessment on the actual status quo of health care for PSS across Europe is necessary to derive broader conclusions on how to foster evidence-based treatment for PSS. This should also include the role of complementary and alternative medicine for PSS, which seems to be widely used but hardly studied.

In conclusion, the gap between evidence-based guidelines and clinical reality needs to be overcome to address individual health burden, to avoid iatrogenic harm and to reduce inappropriate use of health care resources. Therefore, we advocate for more research on the actual European health care situation. A systematic European research agenda with unified goals and interdisciplinary collaboration (13) that integrates all stakeholders (i.e., mental health specialists, PCPs, patients, other relevant somatic specialists) could answer to these challenges.

## Author Contributions

All authors contributed conception and design of the study, organized the expert discussion, and contributed, read, and approved the submitted version. SK and MS-M did the qualitative analysis, conducted the interviews, and drafted the manuscript.

### Conflict of Interest Statement

The authors declare that the research was conducted in the absence of any commercial or financial relationships that could be construed as a potential conflict of interest. The handling Editor declared a past co-authorship with one of the authors BL.

## The EURONET-SOMA Group Includes

Arturs Acans, Gunta Ancane, Marie Bendix, Manfred Beutel, Chris Burton, Paul Enck, Per Fink, Lisbeth Frostholm, Harald Gündel, Peter Henningsen, Paul Hüsing, Chris Kenedi, Ksenya Khohlova, Maria Kleinstäuber, Sebastian Kohlmann, Wijo Kop, Claas Lahmann, Marco Lehmann, Bernd Löwe, Ulrik Frederik Malt, Krzysztof Malyszczak, Alexandra Martin, Arturs Miksons, Tim olde Hartman, Winfried Rief, Judith Rosmalen, Heribert Sattel, Andreas Schröder, Michael Sharpe, Meike Shedden-Mora, Anne Toussaint, Omer van den Bergh, Christina van der Feltz-Cornelis, Angelika Weigel, Ursula Werneke, Michael Witthöft.
